# Hypoxia-Induced Upregulation of lncRNA ELFN1-AS1 Promotes Colon Cancer Growth and Metastasis Through Targeting TRIM14 *via* Sponging miR-191-5p

**DOI:** 10.3389/fphar.2022.806682

**Published:** 2022-05-16

**Authors:** Xu Jing, Lutao Du, Shuang Shi, Aijun Niu, Jing Wu, Yunshan Wang, Chuanxin Wang

**Affiliations:** ^1^ Department of Clinical Laboratory, The Second Hospital of Shandong University, Jinan, China; ^2^ Department of Pharmacy, The Second Hospital of Shandong University, Jinan, China

**Keywords:** ELFN1-AS1, hypoxia, TRIM14, colon cancer, miR-191-5p

## Abstract

Hypoxia is identified as one of the microenvironmental features of most solid tumors and is involved in tumor progression. In the present research, we demonstrate that lncRNA extracellular leucine rich repeat and fibronectin type III domain-containing 1-antisense RNA 1 (ELFN1-AS1) is upregulated by hypoxia in colon cancer cells. Knockdown of ELFN1-AS1 in hypoxic colon cancer cells can reduce cell proliferation and restore the invasion to non-hypoxic levels. Fluorescence in situ hybridization results show that ELFN1-AS1 is distributed in the cytoplasm of colon cancer cells, so we further analyze the potential targets for ELFN1-AS1 as a competing endogenous RNA (ceRNA). MiR-191-5p contains a binding sequence with ELFN1-AS1 and is downregulated by ELFN1-AS1 in colon cancer cells. Then, there is a binding site between miR-191-5p and the 3′ untranslated region of tripartite motif TRIM 14 (TRIM14). The expression of TRIM14 is inhibited by ELFN1-AS1 siRNA or miR-191-5p mimics in LoVo and HT29 cells. The treatment of the miR-191-5p inhibitor in ELFN1-AS1 knockdown cells can significantly increase cell proliferation and invasion ability. Overexpression of TRIM14 in miR-191-5p-mimic-treated cells can rescue the inhibition of proliferation and invasion caused by miR-191-5p mimics. In conclusion, ELFN1-AS1 operates as a downstream target of hypoxia, promotes proliferation and invasion, and inhibits apoptosis through upregulating TRIM14 by sponging miR-191-5p in the colon cancer cells. Our results enrich our understanding of colon cancer progression and provide potential targets for clinical treatment of colon cancer.

## Introduction

Colon cancer is one of the most common malignant tumors in the digestive system, accounting for the third death rate of cancer patients (Siegel et al., 2013). At present, surgery combined with postoperative adjuvant chemotherapy and targeted therapies are mainly used for the treatment of colon cancer, but the therapeutic effect is not satisfactory (Dienstmann et al., 2015). Therefore, it is of great significance to identify and develop new therapeutic targets for colon cancer. Hypoxia is identified as one of the microenvironmental features of most solid tumors (Jing et al., 2019). Moderate hypoxia may be the initiating factor for genetic instability, malignant transformation, and even metastasis of tumor cells (Manoochehri Khoshinani et al., 2016; Jing et al., 2019). Cells that are not sensitive to apoptosis under hypoxic environments are more invasive and resistant to radiotherapy and chemotherapy. Numerous studies have shown that HIF1α plays a central role in the process of tumor adaptation to hypoxia (Manoochehri Khoshinani et al., 2016). However, its mechanism has not been clarified.

In the present research, we found that lncRNA extracellular leucine rich repeat and fibronectin type III domain-containing 1-antisense RNA 1 (ELFN1-AS1) was upregulated by hypoxia in colon cancer cells. In 2014, ELFN1-AS1 was reported for the first time to be generally highly expressed in tumors but low in normal tissues (Polev et al., 2014). Subsequently, through screening for the key lncRNAs in early-stage colon adenocarcinoma (COAD), Liu et al. found that ELFN1-AS1 was associated with early-stage COAD with a potential diagnostic value (Liu et al., 2018). Furthermore, extracellular vesicles (EVs) from human umbilical cord mesenchymal stem cells (hUCMSCs) transfected with ELFN1-AS1-siRNA could inhibit the progression of COAD (Dong et al., 2019). In the latest research, the high expression of ELFN1-AS1 was found to predict poor prognosis in colon cancer through survival analysis based on The Cancer Genome Atlas (TCGA) database (Chen et al., 2020). However, the specific role and mechanism of ELFN1-AS1 in colon cancer remain unclear. In our research, we clarified that hypoxia promoted the growth and metastasis of tumor cells by upregulating the ELFN1-AS1/miR-191-5p/tripartite motif 14 (TRIM14) axis, providing potential targets for the clinical treatment of colon cancer.

## Materials and Method

### Tissue Samples

A total of 32 cases of colon cancer tissues and 32 paired control tissues from patients with colon cancer were collected from the second hospital of Shandong University. The samples were snap-frozen in liquid nitrogen and stored at −80 °C until further analysis. All patients signed informed consents.

### Cell Culture

The human colon cancer cell lines HCT116, SW480, LoVo, and HT29 were maintained in our laboratory. Dulbecco’s modified Eagle’s medium with 0.1 mg/ml streptomycin, 100 U/ml penicillin, and 10% fetal bovine serum (Thermo Fisher Scientific, Shanghai, China) were used for cell culture. The hypoxia model was constructed with 5% CO_2_ and 95% N_2_. Cells in the logarithmic phase were transfected using lipofectamine 2000 (Invitrogen, Carlsbad, CA, United States). ELFN1-AS1 (Transcript: ENST00000415399.1) siRNAs, miR-191-5p mimics, TRIM14 overexpressed plasmids, and scramble sequences were all purchased from RiboBio (Guangzhou, China). The sequences involved in this study are shown as follows: ELFN1-AS1 siRNA, 5′-CCT​TTA​ATC​TCT​TGC​TCA​A-3′; control siRNA, 5′-TTC​TCC​GAA​CGT​GTC​ACG​T-3′; inhibitor control, 5′-GGU​UCG​UAC​GUA​CAC​UGU​UCA-3′; mimic control, 5′-CGG​UAC​GAU​CGC​GGC​GGG​AUA​UC-3′. 50 nM siRNA was used for transfection. Control siRNA, the inhibitor control, the mimic control, and empty plasmid pcDNA3.1 were used as the scramble for ELFN1-AS1 siRNA, the miR-191-5p inhibitor, miR-191-5p mimics, and TRIM14 overexpressed plasmids, respectively.

### Quantitative Reverse Transcription Polymerase Chain Reaction

Total RNA was extracted from LoVo and HT29 cells after the transfection for 24 h using TriQuick Reagent (Solarbio Science and Technology Company, Beijing, China). The cDNA was synthesized using a HiFiScript cDNA Synthesis Kit (CwBio, Beijing, China), and then quantitative reverse transcription polymerase chain reaction (qRT-PCR) was performed using a Fluorescence quantitative PCR kit UltraSYBR Mixture (CwBio, Beijing, China). The primers used in this research were synthesized in GENEWIZ (Suzhou, China). The following primers were used in this research: ELFN1-AS1-F, 5′-GCG​CCT​CAG​CCA​CAA​TCG​TAA​TC-3′; ELFN1-AS1-R, 5′-GGG​GGC​ATG​CAC​CAG​AGG​ACT-3′; TRIM14-F, 5′-TAT​TGC​TGA​AAT​ACG​CGC​GC-3′; TRIM14-R, 5′-GTC​AAC​CTC​CCA​GTA​GTG​GC-3′; Actin-F, 5′-CCC​GAG​CCG​TGT​TTC​CT-3′; Actin-R, 5′-GTC​CCA​GTT​GGT​GAC​GAT​GC-3′. The miRNA Purification Kit (CwBio, Beijing, China), miRNA cDNA Synthesis Kit (CwBio, Beijing, China), and miRNA qPCR Assay Kit (CwBio, Beijing, China) were used for miRNA extraction and detection. The expression of target genes was analyzed using the 2^−ΔΔCT^ method. A qRT-PCR instrument (Funglyn Biotech, Toronto, Canada) was used for the detection. Actin was used as an internal control.

### Western Blot

The protein levels of genes were detected using western blot analysis. After the transfection for 48 h, the protein was extracted using RIPA buffer and separated on 10% sodium dodecyl sulfate–polyacrylamide gel electrolysis gel. Then, the protein was transferred into a PVDF membrane. After 1 h of the block with 5% non-fat milk, the membrane was incubated with primary antibodies at 4°C overnight and subsequently secondary antibodies at room temperature for 1 h. The primary antibodies anti-HIF1α (20960-1-AP) and anti-TRIM14 (15742-1-AP) were purchased from ProteinTech Group (Chicago, IL, United States). Protein bands were developed using an ECL kit (Solarbio Science and Technology Company, Beijing, China).

### Fluorescence *in situ* Hybridization

The distribution of ELFN1-AS1 in HT29 cells was detected by FISH using a Fluorescent *In Situ* Hybridization Kit (RiboBio, Guangzhou, China) following the manufacturer’s manual. The probes of ELFN1-AS1 were designed and synthesized by RiboBio (Guangzhou, China). The fluorescence was observed by using a fluorescence microscope (OLYMPUS, Tokyo, Japan).

### Cell Viability and Proliferation Assays

CCK8 assay was performed to detect cell viability. After transfection, cells were seeded into a 96-well plate at the concentration of 1000 cells/well. Cells were incubated with 10 μl of CCK8 reagent (Solarbio Science and Technology Ltd., Beijing, China) at 37°C for 1.5 h. Cell viability was measured every 24 h. The OD value was detected at 450 nm.

The proliferation of LoVo and HT29 cells was further determined by colony formation assay. After transfection, 500 cells were seeded in a 6-well plate and incubated at 37°C for 14 days. After fixing with 4% paraformaldehyde, cells were stained with GIMSA for 20 min. The photo of plates was taken, and the colony number of each group was counted in five random fields.

### Cell Invasion Assay

The invasive ability of cells was detected using a transwell assay. 100 μl of melted matrigel (Solarbio Science and Technology Ltd., Beijing, China) was added into the upper chamber of the transwell chamber (Invitrogen, Carlsbad, CA, United States) and incubated at 37°C for 4 h. 500 μl of the serum-free medium was added into the lower chamber. Then, 100 μl of the transfected cell suspension (1 × 10^5^ cells, serum-free medium) was added into the upper chamber. After incubation for 24 h, the invasive cells were fixed with 4% paraformaldehyde for 30 min and stained with 0.1% crystal violet for 20 min Five fields of each group were randomly selected and photographed at × 100 magnification. The invasive cells were counted by two independent researchers.

### Cell Apoptosis Analysis With a Flow Cytometer

After 24 h of transfection, cells were resuspended with the binding buffer at a density of 1–5 × 10^6^ cells/ml. 100 μl of the cell suspension was added into a 5 ml flow tube. 5 μl of Annexin V/FITC (4ABio, Beijing, China) was added into the flow tube, which was subsequently incubated at room temperature for 5 min 10 μl of PI solution was added for detection. Flowjo software was used to analyze the flow results.

### Bioinformatics Analysis

The expression and prognosis value of ELFN1-AS1 were obtained through an online TCGA database, GEPIA (http://gepia.cancer-pku.cn/index.html). The differential analysis here is based on 275 colon adenocarcinoma (COAD) and 349 normal tissues. The method for differential analysis is one-way ANOVA. Overall survival analysis was performed using the Log-rank test, a.k.a the Mantel–Cox test, for the hypothesis test.

The sequences of the hypoxic response element (HRE) on the ELFN1-AS1 promoter were predicted on PROMO (http://alggen.lsi.upc.es/cgi-bin/promo_v3/promo/promoinit.cgi?dirDB=TF_8.3). The potential downstream targets of ELFN1-AS1 and miR-191-5p were predicted using Diana tools (DIANA-LncBase v3, https://diana.e-ce.uth.gr/lncbasev3/interactions).

### Luciferase Reporter Assay

The binding status of HRE on the ELFN1-AS1 promoter under hypoxia conditions was detected using a luciferase reporter assay. The sequence containing the wild-type (WT)/mutant-type (MUT) HRE on the ELFN1-AS1 promoter was cloned into the reporter vector (Youbio, Hunan, China). Then, the plasmids carrying WT/MUT HRE were transfected into LoVo and HT29 cells. The luciferase activity was measured using a dual-luciferase assay system (MedChemExpress, Monmouth Junction, United States).

To analyze the interaction between miR-191-5p and ELFN1-AS1 or TRIM14, the wild-type (WT)/mutant-type (MUT) ELFN1-AS1 and WT/MUT TRIM14 3′ untranslated region (UTR) were cloned into the reporter vector (Youbio, Hunan, China), respectively. LoVo cells were co-transfected with miR-191-5p mimics and either of the above vectors. The luciferase activity was measured using a dual-luciferase assay system (MedChemExpress, Monmouth Junction, United States). A microplate reader (TECAN, Grodig, Austria) was used for fluorescence detection.

### Statistical Analysis

SPSS 18.0 statistical analysis software was used to analyze the results. All data were expressed as mean ± standard deviation (SD). A *t*-test was used for comparison between the two groups, and one-way ANOVA was used for the comparison between the multiple groups with Bonferroni as a post hoc test. A paired *t*-test was used to compare 32 pairs of clinical tissues. Pearson correlation analysis was used to analyze the correlation between groups. *p* < 0.05 was statistically significant.

## Results

### ELFN1-AS1 is Highly Expressed in Colon Cancer Tissues and Cells

**FIGURE 1:**
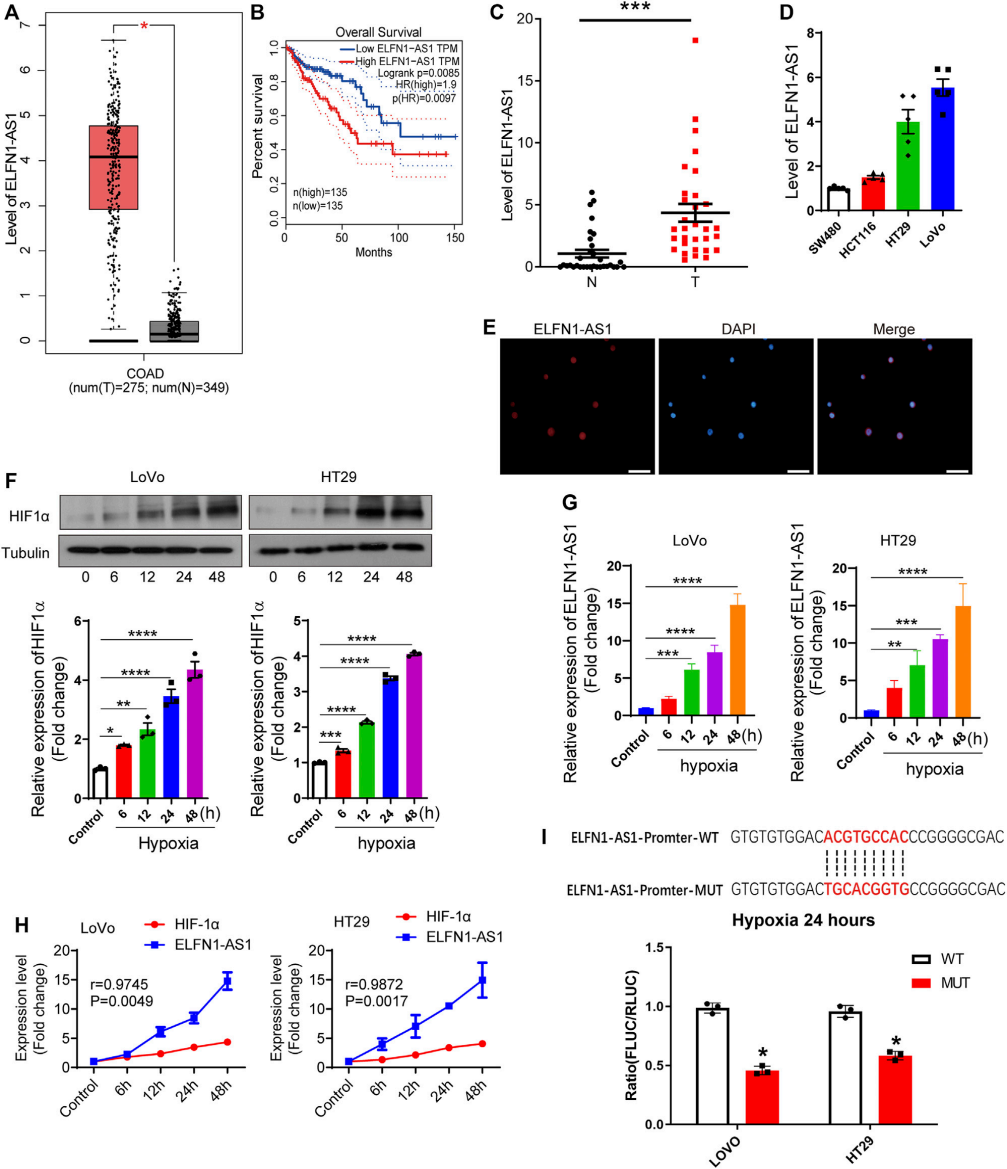
Hypoxia induces overexpression of ELFN1-AS1 in colon cancer. **(A)** Expression level of ELFN1-AS1 in colon adenocarcinoma (COAD) was analyzed on the TCGA database (GEPIA). Compared with the normal tissues (*n* = 349), ELFN1-AS1 was markedly upregulated in COAD tissues (*n* = 275) (*p* < 0.01). The method for differential analysis is one-way ANOVA. **(B)** Prognosis value of ELFN1-AS1 in COAD was analyzed on the TCGA database (GEPIA). The COAD tissues were divided into two groups (low ELFN1-AS1 and high ELFN1-AS1 groups) at a 50% cutoff. Overall survival analysis was performed using the Log-rank test, a.k.a the Mantel–Cox test, for the hypothesis test. **(C)** qRT-PCR was performed to detect the expression of ELFN1-AS1 in 32 pairs of clinical tissues of colon cancer collected from local hospitals. Actin was used as an internal control. A paired *t*-test was used to compare 32 pairs of clinical tissues. **(D)** qRT-PCR was used to determine the ELFN1-AS1 level in four human colon cancer cell lines, HCT116, SW480, HT29, and LoVo. **(E)** Expression and distribution of ELFN1-AS1 in HT29 cells were analyzed by FISH. The red fluorescence and blue fluorescence represent ELFN1-AS1 and the nucleus, respectively. Scale bar 10 μm. **(F)** After 0, 6, 12, 24, and 48 h of hypoxia, the expression of HIF1α was detected using western blot. **(G)** After 0, 6, 12, 24, and 48 h of hypoxia, the expression of ELFN1-AS1 was detected using RT-PCR in LoVo and HT29 cells. **(H)** Correlation between ELFN1-AS1 and HIF-1α was analyzed using Pearson correlation analysis. **(I)** Binding status of HRE on the ELFN1-AS1 promoter under hypoxia conditions was detected using a dual luciferase reporter assay. One-way ANOVA was used for the comparison between the multiple groups with Bonferroni as a post hoc test. All data were representative of three independent experiments. *p < 0.05; ***p < 0.001; ****p < 0.0001.

First, the expression and prognosis value of ELFN1-AS1 were obtained through a TCGA database, GEPIA (Tang et al., 2017). As shown in Figure 1A, ELFN1-AS1 was markedly upregulated in COAD tissues (*n* = 275) compared with the normal tissues (*n* = 349) (*p* < 0.01). The prognosis of COAD patients with low ELFN1-AS1 levels was better than that of high-ELFN1-AS1 patients (*p* = 0.0085, Figure 1B). A total of 32 pairs of clinical tissues of colon cancer were collected from the local hospital, and the ELFN1-AS1 level was detected using qRT-PCR. Our results showed that ELFN1-AS1 was significantly upregulated in colon cancer tissues compared with para-cancerous tissues (*p* < 0.001, Figure 1C). Then, the ELFN1-AS1 level was detected in four human colon cancer cell lines, HCT116, SW480, HT29, and LoVo. As shown in Figure 1D, the two cell lines with high ELFN1-AS1 levels (HT29 and LoVo) were used for subsequent experiments. Next, the distribution of ELFN1-AS1 in HT29 cells was analyzed by FISH assay. As shown in Figure 1E, ELFN1-AS1 was expressed in both the cytoplasm and nucleus, suggesting that ELFN1-AS1 has the potential to function as a competing endogenous RNA (ceRNA) in colon cancer cells.

### Hypoxia Upregulates ELFN1-AS1 in Colon Cancer Cells

Subsequently, we investigated the relationship between hypoxia and ELFN1-AS1 expression. LoVo and HT29 cells were incubated at 37°C with 5% CO_2_ and 95% N_2_ to construct a hypoxia cell model. As shown in Figure 1F **,** HIF1α expression gradually increased in a time-dependent manner in both LoVo and HT29 cells. Then, the expression of ELFN1-AS1 in LoVo and HT29 cells was detected after 24 h of hypoxia using qRT-PCR. As shown in Figure 1G, ELFN1-AS1 was significantly upregulated in LoVo and HT29 cells under hypoxia compared with that in routine culture in a time-dependent manner (*p* < 0.001). In addition, Pearson correlation analysis showed that there was a significant positive correlation between the expressions of HIF1α and ELFN1-AS1 in hypoxic LoVo and HT29 cells (Figure 1H). The binding status of HRE on the ELFN1-AS1 promoter under hypoxia conditions was detected using a dual luciferase reporter assay. The sequences of HRE on the ELFN1-AS1 promoter were predicted on PROMO (http://alggen.lsi.upc.es/cgi-bin/promo_v3/promo/promoinit.cgi?dirDB=TF_8.3). Plasmids carrying wild and mutant elements were transfected into colon cancer cells. As shown in Figure 1I, after 24 h of hypoxia, the fluorescence ratio of the MUT group was significantly lower than that of the WT group, suggesting that hypoxia promoted ELFN1-AS1 transcription through HRE. These results indicated that ELFN1-AS1 might be a downstream target of hypoxia response in colon cancer cells.

### ELFN1-AS1 is a Downstream Target of Hypoxia Response in Colon Cancer Cells

**FIGURE 2:**
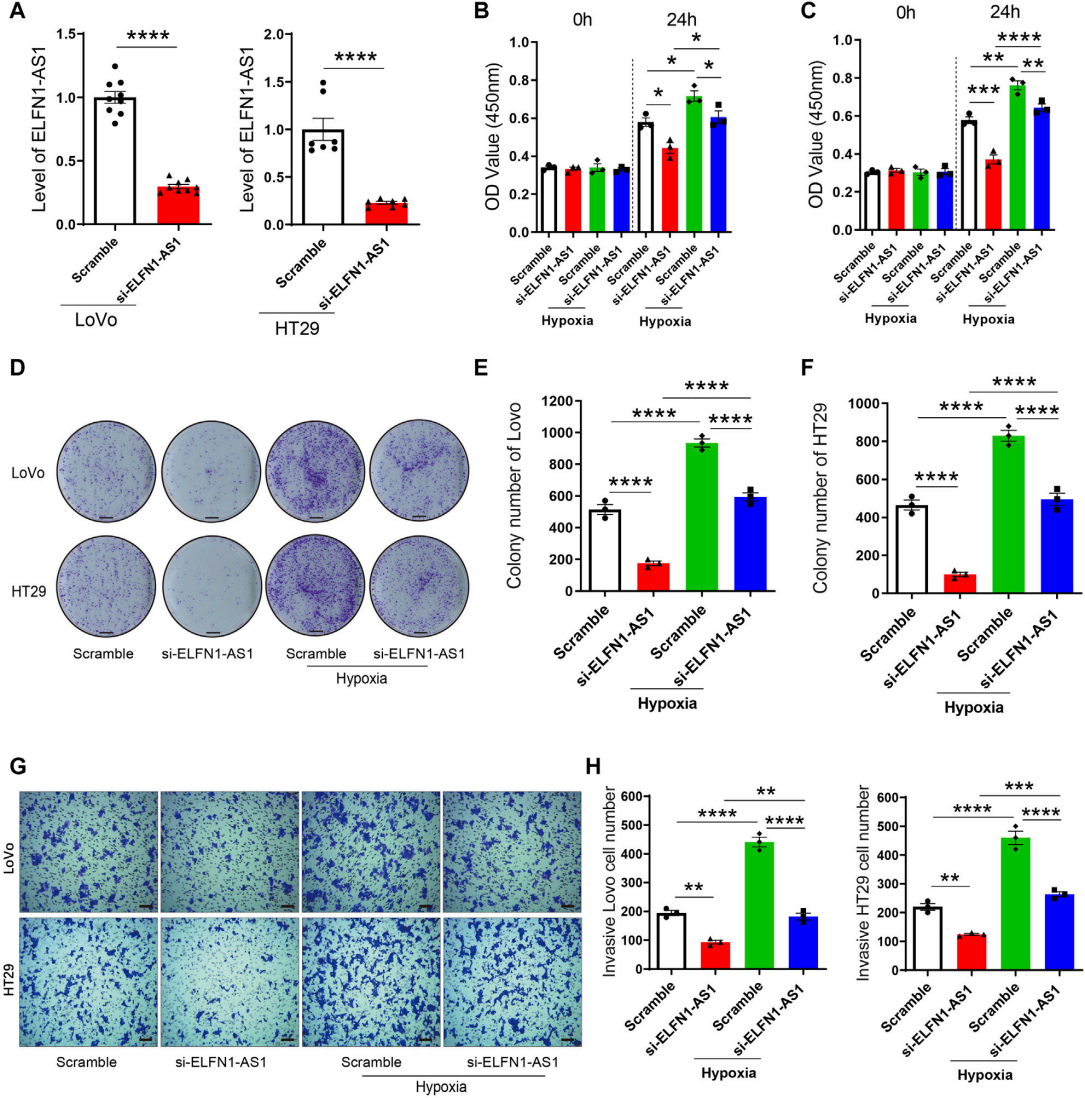
ELFN1-AS1 rescues the proliferation and invasion induced by hypoxia in colon cancer cells. **(A)** ELFN1-AS1-specific siRNAs were transfected into LoVo and HT29 cells with the negative siRNA as a control (Scramble). The ELFN1-AS1 level was detected using qRT-PCR after the transfection for 24 h. A *t*-test was used for this comparison. **(B)** and **(C)** After transfection with ELFN1-AS1 siRNA and empty plasmids, LoVo and HT29 cells were cultured in 5% CO_2_ and 95% N_2_ to generate the hypoxia + scramble and hypoxia + siELFN1-AS1 groups. Cell viability was detected using CCK8 assay. **(D)** Proliferation of LoVo and HT29 cells was determined using a colony formation assay. The photo of the plates was taken after 14 days of incubation. **(E)** and **(F)** Colony number of each group was counted in five random fields. Scale bar 5 mm. **(G)** Transwell assay was performed to detect the invasion of each group. After 24 h of culture in matrigel chambers, cells were photographed under a microscope at ×100 magnification. Scale bar 100 *µ*. **(H)** Number of invasive cells was counted in three random fields. One-way ANOVA was used for the comparison between the multiple groups with Bonferroni as a post hoc test. All data were representative of three independent experiments. *p < 0.05; **p < 0.01; ***p < 0.001; ****p < 0.0001.

According to our results, hypoxia induced the expression of ELFN1-AS1 in LoVo and HT29 cells, suggesting that ELFN1-AS1 may be a downstream factor of hypoxia response. To verify this hypothesis, LoVo and HT29 cells transfected with ELFN1-AS1 siRNA or negative siRNA were incubated under hypoxic conditions (5% CO_2_ and 95% N_2_) to generate the hypoxia + scramble and hypoxia + siELFN1-AS1 groups, respectively. As shown in Figure 2A, siRNA could significantly inhibit the expression of ELFN1-AS1 in both LoVo and HT29 cells compared with the scramble group. Compared with the scramble cells, the cell viability of the hypoxia group was enhanced significantly in both LoVo and HT29 cells. Interestingly, the OD value of LoVo and HT29 cells declined significantly after the transfection of ELFN1-AS1 siRNA under hypoxic conditions (Figures 2B,C). The result of the colony formation assay was consistent with CCK8’s results. Knockdown of ELFN1-AS1 significantly decreased the colony number in LoVo cells; the colony numbers of LoVo and HT29 cells cultured under hypoxic conditions declined after the transfection of ELFN1-AS1 siRNA (Figures 2D–F). In addition, the invasive cell number of LoVo and HT29 cells transfected with ELFN1-AS1 siRNA decreased significantly compared with that of control siRNA; hypoxia increased the invasive cell number, while knockdown of ELFN1-AS1 decreased the invasive cell number to a normal level in LoVo and HT29 cells (Figures 2G,H). These results proved that knockdown of ELFN1-AS1 blocked the proliferation and invasion induced by hypoxia in colon cancer cells.

### Knockdown of ELFN1-AS1 Induces the Apoptosis in Hypoxic Colon Cancer Cells

**FIGURE 3:**
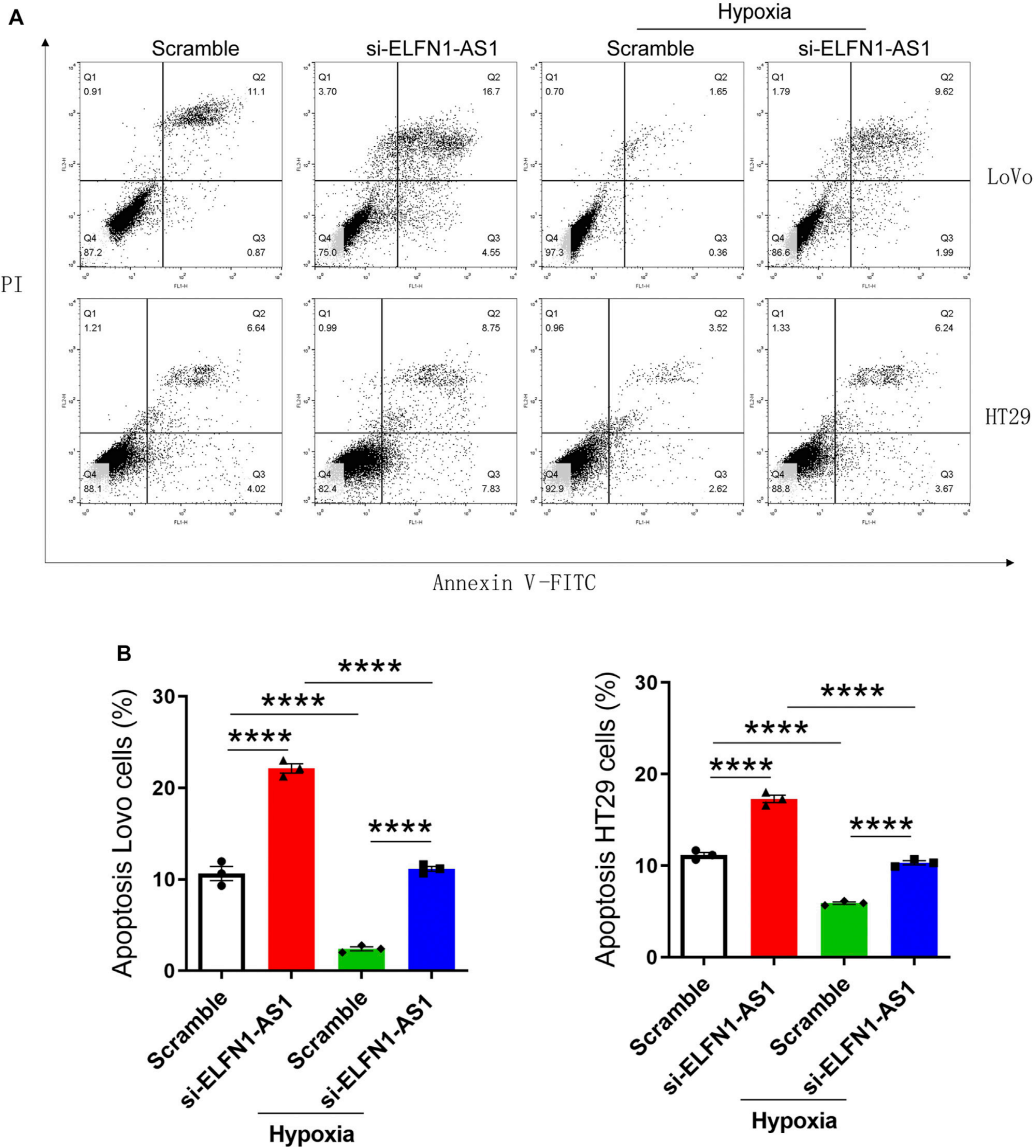
ELFN1-AS1 blocks the inhibition of apoptosis induced by hypoxia in colon cancer cells. **(A)** Flow cytometry analysis was performed to detect the apoptosis in each group after the transfection for 24 h. **(B)** Percentage of apoptotic cells was analyzed using Flowjo software. Analysis was performed using one-way ANOVA with Bonferroni as a post hoc test. All data were representative of three independent experiments. ****p < 0.0001.

Next, the apoptosis of LoVo and HT29 cells was detected using flow cytometry analysis. As shown in Figure 3, the percentage of apoptotic cells significantly increased after the transfection of ELFN1-AS1 siRNA in LoVo cells; in HT29 cells, ELFN1-AS1 siRNA also increased the percentage of apoptotic cells compared with the control siRNA. Our data indicated that knockdown of ELFN1-AS1 induced the apoptosis in colon cancer cells.

We further analyzed the apoptosis of LoVo and HT29 cells cultured under hypoxic conditions. As shown in Figure 3, after 24 h of hypoxia culturing, the apoptosis of LoVo and HT29 cells was significantly inhibited. However, knockdown of ELFN1-AS1 raised the percentage of apoptotic cells to a normal level in both hypoxic LoVo and HT29 cells. These results proved that ELFN1-AS1 knockdown rescued hypoxia-induced inhibition of apoptosis in colon cancer cells.

### ELFN1-AS1 Promotes the Expression of TRIM14 Through Sponging miR-191-5p

**FIGURE 4:**
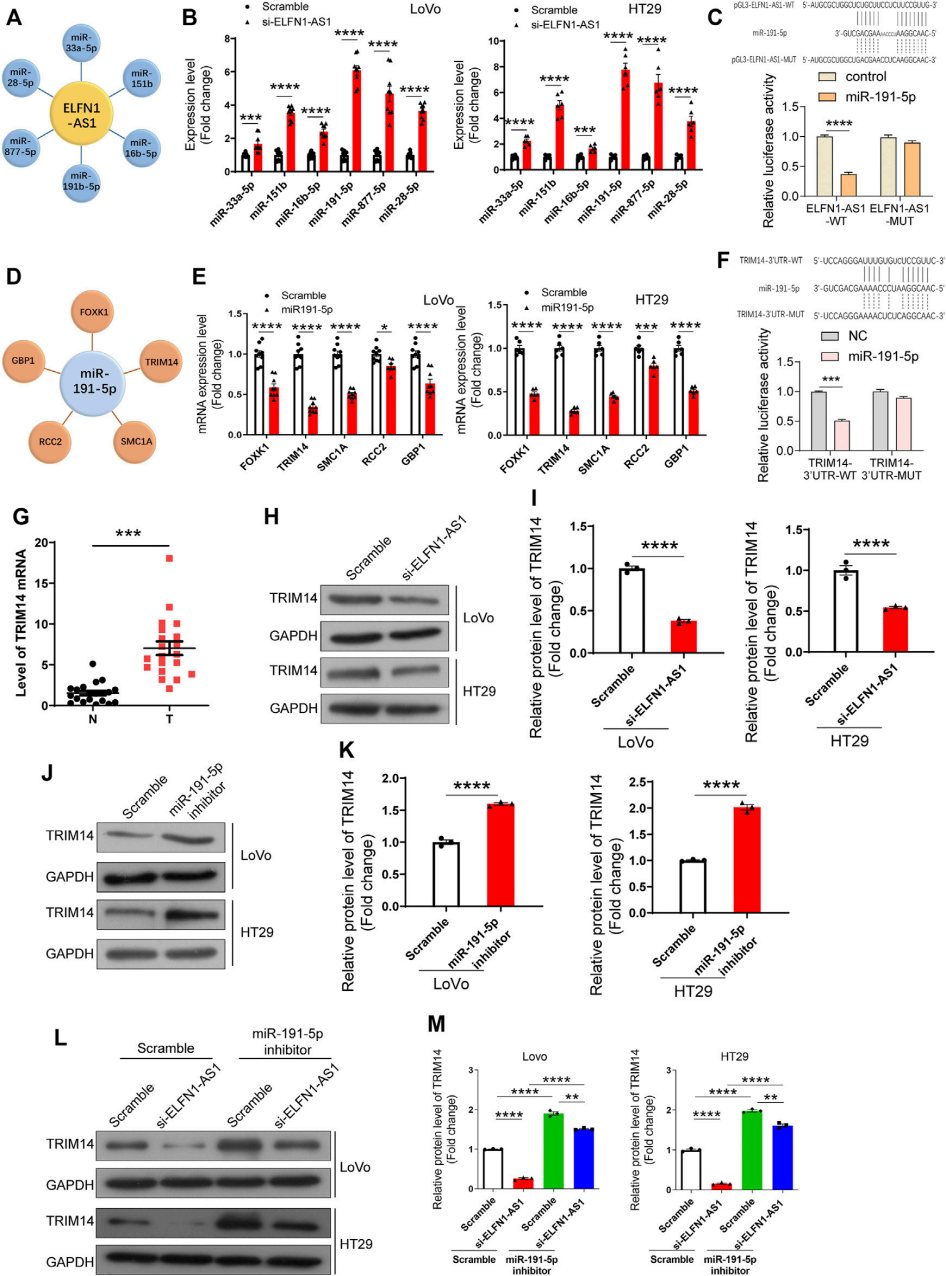
Analysis and verification of ELFN1-AS1’s downstream targets in colon cancer cells. **(A)** miRNAs with potential binding sites to ELFN1-AS1 were predicted in Diana tools (https://diana.e-ce.uth.gr/lncbasev3/interactions). **(B)** Six miRNAs (miR-33a-5p, miR-151b, miR-16b-5p, miR-191-5p, miR-877-5p, and miR-28-5p) were detected in ELFN1-AS1 knockdown cells using qRT-PCR. **(C)** Interaction between miR-191-5p and ELFN1-AS1 was detected using luciferase reporter assay. **(D)** Potential downstream targets of miR-191-5p were also analyzed in Diana tools. **(E)** Five genes (FOXK1, TRIM14, SMC1A, RCC2, and GBP1) were verified in HT29 cells transfected with miR-191-5p mimics. **(F)** Interaction between miR-191-5p and TRIM14 was detected using luciferase reporter assay. **(G)** mRNA level of TRIM14 was detected in 32 pairs of clinical tissues of colon cancer using RT-PCR. A paired *t*-test was used for data analysis. **(H)** and **(I)** Protein level of TRIM14 was detected using western blot after the transfection of si-ELFN1-AS1. **(J)** and **(K)** Protein level of TRIM14 in miR-191-5p knockdown cells was detected using western blot. **(L)** and **(M)** Expression level of TRIM14 in ELFN1-AS1 and miR-191-5p knockdown cells was detected using western blot. A *t*-test was used for comparison between the two groups, and one-way ANOVA was used for the comparison between the multiple groups with Bonferroni as a post hoc test. All data were representative of three independent experiments. **p < 0.01; ***p < 0.001; ****p < 0.0001.

ELFN1-AS1 is abundantly distributed in the cytoplasm, suggesting that it may operate as a ceRNA to regulate the expression of downstream targets. Therefore, we predicted the potential downstream targets of ELFN1-AS1 using Diana tools (https://diana.e-ce.uth.gr/lncbasev3/interactions) (Paraskevopoulou et al., 2016). Analysis results showed that there were six miRNAs which contained potential binding sites of ELFN1-AS1, namely, miR-33a-5p, miR-151b, miR-16b-5p, miR-191-5p, miR-877-5p, and miR-28–5p (Figure 4A). Then, the expression levels of six potential miRNAs were detected using qRT-PCR in LoVo and HT29 cells. As shown in Figure 4B, after ELFN1-AS1 knockdown, the expression of five miRNAs increased significantly (miR-33a-5p, miR-151b, miR-191-5p, miR-877-5p, and miR-28-5p), of which miR-191-5p showed the most significant upward trend. Then, we analyzed the interaction between miR-191-5p and wild or mutant ELFN1-AS1 using the double luciferase reporter gene. As shown in Figure 4C, miR-191-5p could interact with ELFN1-AS1, which was weakened when the predicted binding site was mutated.

We subsequently analyzed the downstream targets of miR-191-5p. The results showed that there were five genes’ 3 ‘UTR regions (FOXK1, TRIM14, SMC1A, RCC2, and GBP1) in which there were binding sites of miR-191-5p (Figure 4D). The expression of FOXK1, TRIM14, SMC1A, and GBP1 declined significantly in LoVo and HT29 cells transfected with miR-191-5p mimics (Figure 4E). TRIM14 showed the most significant downward trend. The combination of miR-191-5p and TRIM14 was confirmed using double luciferase reporter gene analysis. As shown in Figure 4F, miR-191-5p could bind to wild-type TRIM14, while mutations at the predicted site in TRIM14 3′UTR significantly weaken this interaction. Importantly, the mRNA level of TRIM14 was significantly upregulated in 32 colon cancer tissues compared with para-cancerous tissues (Figure 4G).

These results suggested that ELFN1-AS1 upregulated the expression of TRIM14 by sponging miR-191-5p in colon cancer cells.

To verify our hypothesis, we further explored the effect of ELFN1-AS1 and miR-191-5p on TRIM14 expression. As shown in Figures 4H,I, the protein level of TRIM14 declined significantly in ELFN1-AS1 knocked-down LoVo and HT29 cells. After the treatment with the miR-191-5p inhibitor, the protein level of TRIM14 increased significantly (Figures 4J,K). The expression of TRIM14 in ELFN1-AS1 and miR-191-5p knockdown cells increased significantly compared with that of ELFN1-AS1 knockdown cells, while the expression of TRIM14 in ELFN1-AS1 and miR-191-5p knockdown cells increased significantly compared with that of miR-191-5p knockdown cells (Figures 4L,M).

TRIM14 rescues the inhibition of the proliferation and invasion induced by ELFN1-AS1 knockdown in colon cancer cells.

**FIGURE 5:**
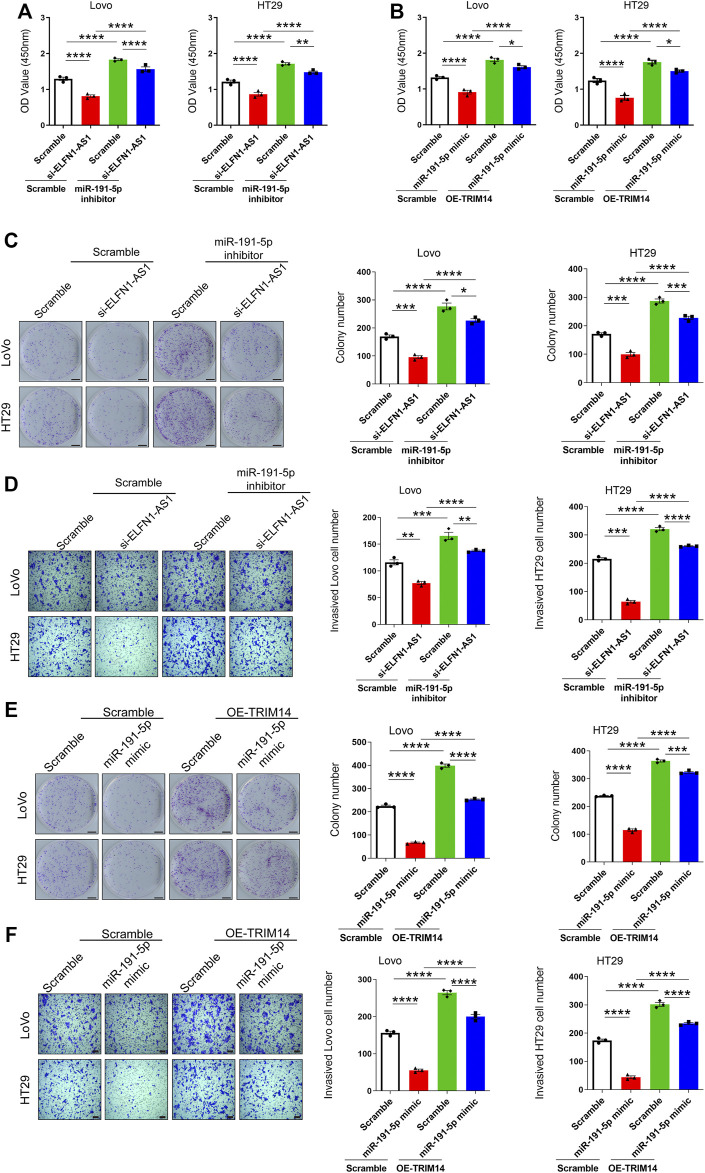
Knockdown of ELFN1-AS1 inhibited the proliferation and invasion by regulating the expression of TRIM14 by targeting miR-191-5p. **(A)** and **(B)** Cell viability was detected using CCK8 assay. **(C)** Colony formation assay was performed to determine the proliferation of LoVo and HT29 cells. The photo of the plates was taken after 14 days of incubation. The colony number of each group was counted in five random fields. Scale bar 5 mm. **(D)** Transwell assay was performed to detect the invasion of each group. After 24 h of culture in matrigel chambers, cells were photographed under a microscope at ×100 magnification. The number of invasive cells was counted in three random fields. Scale bar 100 *µ*. **(E)** Colony formation assay was performed to determine the proliferation of LoVo and HT29 cells. Scale bar 5 mm. **(F)** Transwell assay was performed to detect the invasion of each group. Scale bar 100 *µ*. One-way ANOVA was used for the comparison between the multiple groups with Bonferroni as a post hoc test. All data were representative of three independent experiments. *p < 0.05; **p < 0.01; ***p < 0.001; ****p < 0.0001.

According to the above results, ELFN1-AS1 may promote the expression of TRIM14 by sponging miR-191-5p and finally involve in the regulation of tumor cell behavior. We performed rescue experiments to confirm this hypothesis. As shown in Figure 5A, the miR-191-5p inhibitor promoted cell viability in both LoVo and HT29 cells. After 24 h of simultaneous knockdown of ELFN1-AS1 and miR-191-5p, cell viability increased significantly compared with that of only ELFN1-AS1 knockdown cells (Figure 5A). As shown in Figure 5B, TRIM14 overexpression significantly increased the OD450 value; cell viability in TRIM14 overexpression plus miR-191-5p-mimic-treated cells increased significantly compared with that of miR-191-5p-mimic-treated cells. Compared with that of TRIM14 overexpression cells, cell viability declined significantly in TRIM14 overexpression plus miR-191-5p-mimic-treated cells.

miR-191-5p knockdown increased the proliferation. After the simultaneous knockdown of ELFN1-AS1 and miR-191-5p, the proliferation of LoVo and HT29 cells increased compared with that of only ELFN1-AS1 knockdown cells (Figure 5C). A similar trend was observed in the transwell assay. The invasive cell number in the knockdown of ELFN1-AS1 and miR-191-5p cells increased significantly compared with that of only ELFN1-AS1 knockdown cells and declined significantly compared with that of only miR-191-5p knockdown cells (Figure 5D).

TRIM14 overexpression inhibited the proliferation in miR-191-5p-mimic-treated cells (Figure 5E). The invasive cell number in TRIM14 overexpression plus treatment of miR-191-5p-mimic cells increased significantly compared with that of miR-191-5p-mimic-treated cells and declined significantly compared with that of TRIM14 overexpression cells (Figure 5F). These results showed that the treatment of the miR-191-5p inhibitor in ELFN1-AS1 knockdown cells can significantly increase cell proliferation and invasion ability, and overexpression of TRIM14 in miR-191-5p-mimic-treated cells can rescue the inhibition of proliferation and invasion caused by miR-191-5p mimics.

## Discussion

The hypoxic microenvironment widely exists in solid tumors, which is mainly due to the increased oxygen consumption and decreased oxygen supply caused by microenvironmental obstacles and abnormal functions of tumor blood vessels (Patel and Sant, 2016). In colon cancer, hypoxia induces the production of vascular endothelial growth factor (VEGF), which initiates tumor angiogenesis (Kim et al., 2015; Kawczyk-Krupka et al., 2018). However, due to the abnormality structure of the blood vessels, the tumor hypoxia persists. In the hypoxic microenvironment, colon cancer cells undergo a series of biochemical changes in response to hypoxia, including enhanced anaerobic glycolysis and increased protective stress proteins, which include specific cytokines and growth factors, such as erythropoietin, VEGF glycolytic enzymes, and transcription factors AP-1, NFKB, and HIF1 (Vasilevskaya et al., 2008; Gombos et al., 2011; Ni et al., 2017; Vadde et al., 2017). These factors regulate the function of colon cancer cells by regulating the expression of downstream targets (Costa et al., 2017; Chen et al., 2018a). For example, HIF1-α activates p21 transcription, which blocks the interaction between VHL and HIF1 α, then inhibits the interpretation of HIF1 α, forms a positive feedback regulatory loop, and finally promotes cell growth of colon cancer (Yang et al., 2014).

In the present research, we found that cell hypoxia significantly increased the expression of ELFN1-AS1. Hypoxia promoted proliferation and invasion and inhibited the apoptosis of colon cancer cells. Knocking down ELFN1-AS1 under hypoxic conditions significantly reduced cell proliferation and invasion and increased apoptosis significantly. The specificity and limited dosage of ELFN1-AS1 siRNA avoided the apoptosis caused by excessive siRNA mediated off-target effects. In addition, knockdown of ELFN1-AS1 in conventionally cultured colon cancer cells effectively inhibited cell growth and invasion. These results indicate that ELFN1-AS1 may be a promoter of abnormal proliferation of colon cancer cells and operates as a target in the hypoxic response of tumor cells. At present, ELFN1-AS1 has been found to be upregulated in esophageal cancer and facilitates esophageal cancer progression (Zhang et al., 2019). It is also reported to be highly expressed in colon cancer and can predict the prognosis of patients (Dong et al., 2019; Chen et al., 2020). However, our understanding of the role and mechanism of ELFN1-AS1 in tumors is still limited.

We found that ELFN1-AS1 was distributed in the cytoplasm and nucleus of colon cancer cells, suggesting that it might operate as ceRNA in the cytoplasm. CeRNAs regulate gene expression by competing with miRNAs and are widely involved in the regulation of tumor growth, metastasis, recurrence, and drug resistance (Salmena et al., 2011; Qi et al., 2015; Wang et al., 2016). In the present study, we demonstrated that ELFN1-AS1, as a ceRNA, competitively adsorbed miR-191-5p to promote the expression of TRIM14 through bioinformatics analysis and cell line verification.

According to current research, miR-191-5p exerts a tumor-suppressive role in lung adenocarcinoma, COAD, and renal cell carcinoma (Chen et al., 2018b; Chen et al., 2018c; Zhou et al., 2020). Chen et al. report that the miR-191-5p level is negatively associated with PD-L1 expression and predicts overall survival (OS) as an independent prognostic factor in COAD (Chen et al., 2018b). However, the downstream targets of miR-191-5p in colon cancer have not been reported. According to our research, overexpression of miR-191-5p inhibited the expression of TRIM14 in LoVo and HT29 cells. TRIM14, as a member of the trim family, has been proved to be upregulated in breast cancer, hepatocellular carcinoma, and oral squamous cell carcinoma and can regulate tumor biological behavior through a variety of signaling pathways (Chen et al., 2016; Wang et al., 2017a; Dong and Zhang, 2018; Hu et al., 2019). Studies have shown that TRIM14 can be regulated by different miRNAs in different tumors. For example, TRIM14 is inhibited by miR-15b and enhances cancer-initiating cell phenotypesoral in tongue squamous cell cancer (Wang et al., 2017b). MiR-195-5p can downregulate the expression of TRIM14 and then promote the progression of gastric cancer by regulating the epithelial–mesenchymal transition process (Wang et al., 2018). In a recent study, TRIM14 is reported to promote migration and invasion by regulating sphingosine kinase 1 (SPHK1) and phosphorylated STAT3 (p-STAT3) in CRC cells (Jin et al., 2018). However, due to the limitations of the experiment, the demonstration of direct ceRNA interactions in this study is limited to a single gene. In a huge ceRNA network, a single lncRNA or miRNA can target numerous genes. Therefore, other miRNAs and genes detected in this study may be potential targets of ELFN1-AS1, and their role remains to be further studied.

In addition, our results showed that HIF1 α was overexpressed under hypoxia in a time-dependent manner. HIF1 α is the main mediator of cell adaptation to hypoxia. In the hypoxic environment, it can enhance the transcription of hypoxia-induced genes and produce a series of physiological effects (Soni and Padwad, 2017; Pezzuto and Carico, 2018). However, we did not explore the effect of intermediate targets of HIF1 α on the resulting ELFN1-AS1/miR-191-5p/TRIM14 pathway, which will be our interest in further study.

In conclusion, ELFN1-AS1 operates as a downstream target of hypoxia to promote proliferation and invasion through upregulating TRIM14 by sponging miR-191-5p in colon cancer cells. Our results enrich the regulatory network of ceRNA and provide potential targets for the clinical treatment of colon cancer.

## Data Availability

The original contributions presented in the study are included in the article/Supplementary Material, further inquiries can be directed to the corresponding author.
